# Hypertonic saline attenuates the cytokine-induced pro-inflammatory signature in primary human lung epithelia

**DOI:** 10.1371/journal.pone.0189536

**Published:** 2017-12-18

**Authors:** Sanchayita Mitra, Daran Schiller, Cameron Anderson, Fabia Gamboni, Angelo D’Alessandro, Margeurite Kelher, Christopher C. Silliman, Anirban Banerjee, Kenneth L. Jones

**Affiliations:** 1 Department of Surgery/Trauma Research Center, University of Colorado Denver, School of Medicine, Aurora, Colorado, United States of America; 2 Department of Biochemistry and Molecular Genetics, University of Colorado Denver, School of Medicine, Aurora, Colorado, United States of America; 3 Bonfils Blood Center, Denver, Colorado, United States of America; 4 Dept. of Pediatrics, University of Colorado Denver, School of Medicine, Aurora, Colorado, United States of America; Hospital for Sick Children, CANADA

## Abstract

Trauma/hemorrhagic shock is a complex physiological phenomenon that leads to dysregulation of many molecular pathways. For over a decade, hypertonic saline (HTS) has been used as an alternative resuscitation fluid in the setting of trauma/hemorrhagic shock. In addition to restoring circulating volume within the vascular space, studies have shown a positive immunomodulatory effect of HTS. Targeted studies have shown that HTS affects the transcription of several pro-inflammatory cytokines by inhibiting the NF-κB–IκB pathway in model cell lines and rats. However, few studies have been undertaken to assess the unbiased effects of HTS on the whole transcriptome. This study was designed to interrogate the global transcriptional responses induced by HTS and provides insight into the underlying molecular mechanisms and pathways affected by HTS. In this study, RNA sequencing was employed to explore early changes in transcriptional response, identify key mediators, signaling pathways, and transcriptional modules that are affected by HTS in the presence of a strong inflammatory stimulus. Our results suggest that primary human small airway lung epithelial cells (SAECS) exposed to HTS in the presence and absence of a strong pro-inflammatory stimulus exhibit very distinct effects on cellular response, where HTS is highly effective in attenuating cytokine-induced pro-inflammatory responses via mechanisms that involve transcriptional regulation of inflammation which is cell type and stimulus specific. HTS is a highly effective anti-inflammatory agent that inhibits the chemotaxis of leucocytes towards a pro-inflammatory gradient and may attenuate the progression of both the innate and adaptive immune response.

## Introduction

Trauma/hemorrhagic shock is a leading cause of death in people under the age of 44 in the United States [1]. Acute lung injury (ALI) and subsequent acute respiratory distress syndrome (ARDS) were first described by Ashbaugh and Petty in 1967, and remain a significant cause of morbidity and mortality in patients that survive the initial phase of shock [2] with mortality estimated at 24% [3]. The acute inflammatory response starts with the firm adhesion of polymorphonuclear neutrophils (PMNs) to the pulmonary vascular endothelium followed by extravasation of these PMNs into the alveolar spaces through the damaged endothelium and initiation of a self-propagative inflammatory cascade [4]. This cascade also recruits other immune cells to amplify this response, releases inflammatory mediators into the systemic circulation, and triggers a systemic inflammatory response resulting in multi-organ failure and increased mortality in ARDS patients [4, 5].

Epithelial cells are critical to the pathogenesis of ALI/ARDS, because they provide a surface for gas exchange, function as a barrier to external pathogens, and play an important role in the recognition and resolution of inflammatory responses inherent to ALI [6, 7]. Epithelial cells express a wide variety of immunomodulatory molecules in response to inflammatory challenge [7] and can regulate leucocyte influx into the alveolar space through the production of pro-inflammatory mediators [8, 9]. While some of this inflammatory response is required to maintain homeostasis, a hyperinflammatory response can have negative consequences and may cause fibrosis and tissue damage by the release of proteases such as neutrophil elastase [10], matrix metalloproteases [11–13] and generation of reactive oxygen intermediates [7, 10]. Thus, it is critical to dampen or inhibit the hyperinflammatory response to prevent the progression of ARDS.

HTS is currently used as an intravenous resuscitation fluid in the setting of trauma and hemorrhagic shock and has been associated with improved clinical outcomes [14]. In addition to restoring plasma volume within the vascular space, beneficial immunomodulatory effects are observed in animal models where intravenous HTS resuscitation effectively reduced lung inflammation and attenuated ALI [15–20]. However intravascular HTS administration may change the osmolality of circulating blood and produce other undesirable systemic side effects. Nebulizing HTS to the lungs would avoid these deleterious effects without affecting its immunomodulatory effects in the organ where this effect is most needed in the setting of ARD/ALI. Additionally, in a rat model, these immunomodulatory effects were observed when delivered to the apical membrane of epithelial cells through aerosol administration. When given immediately following hemorrhagic shock, nebulized HTS attenuated lung injury and was also able to inhibit the synthesis of pro-inflammatory chemokines [21]. Currently an ongoing phase I clinical trial at the Denver Health Medical Center (NCT01667666) is investigating the therapeutic effects of nebulized HTS administration in patients with ARDS secondary to trauma. Our group has previously reported the anti-inflammatory effects of HTS in an in-vitro model using the transformed A549 type II pulmonary lung epithelial cells [8, 9]. Despite these valuable data, the effect of HTS on primary human small airway respiratory epithelium and its role in decreasing ALI are poorly understood. Further, A549 cells demonstrate aneuploidy, aberrant mitosis, and do not display contact inhibition; thus, such transformed cells have limited clinical utility and likely display signaling that is different from primary human cells. In the absense of commercial source of Type I and Type II pneumocytes (the major mediators in ARDS), we used primary pulmonary SAECS for our studies. SAECS are primary human cells isolated from the distal 1mm area of human bronchioles and represent a better model to interrogate the underlying molecular mechanisms driving the anti-inflammatory effects of HTS on the inflamed lung epithelial cell. In the current study, RNA sequencing was employed to yield valuable insights about global transcriptional changes of the respiratory epithelium following a pro-inflammatory challenge and subsequent HTS treatment. If HTS proves to be effective in the reduction of inflammation in the lung epithelium, nebulization could be used as a valuable treatment strategy for the treatment of ALI and prevent changes in osmolality of circulating blood by intravenous administration, which can cause undesirable systemic effects.

## Materials and methods

### Cell culture and stimulation

Primary human small airway epithelial cells (SAECS, Lonza Bioproducts, Allendale NJ) in P2 were grown to 80% confluence in Ti 75 flasks in a humidified chamber with 5% CO_2_ in small airway growth medium containing growth supplements (SAGM media, Lonza Bioproducts) and re-seeded into 6, 12, or 24-well plates depending on the experimental requirement. 400mOsm HTS media was prepared by supplementing 100mL SAGM media with 1.55mL of 23.4% clinical grade HTS (APP Pharmaceuticals, LLC Schaumburg, IL) obtained from the University Pharmacy. All experiments were performed with cells in Passage 3 (P3) or Passage 4 (P4) at 80% cell confluence, using cells from different donors (i.e., different lots) for each experiment. As a combination of TNF-α, IL-β and IFN-γ have been shown to act synergistically in the pathogenesis of ALI [22], we used a cocktail of these cytokines as a pro-inflammatory challenge in this study. The following 4 conditions were tested using 3 replicates each: control (untreated), cytomix-treated [cytomix; TNF-α 10n/mL, IL-1β 10ng/mL (Sigma Aldrich, St Louis, Mo) and IFN-γ 10ng/mL (R&D Biosystems, Minneapolis, MN)], HTS (400mOsm hypertonic saline) and HTS+cytomix. IRF1 antibody (Santa Cruz Biotechnology, Inc.). Total STAT1 and p-STAT1 antibodies (Cell Signaling Technologies) and β-Tubulin antibody (BD biosciences) were used for western blots. 400mOsm hypertonic saline was chosen as the treatment dose because this dose of HTS has been used as a resuscitative fluid to restore mean arterial pressure and microvascular circulation in the setting of trauma without significant adverse effects and also elicits an effective anti-inflammatory response [19, 20, 23] (S1 Fig). We also show that 400mOsm is the minimal dose necessary to obtain a strong anti-inflammatory response.

### RNAseq and data analysis

Total RNA from P3 SAECS was isolated using Qiagen RNeasy miniprep kit (Qiagen Inc., Germantown, MD). Final RNA preparations were suspended in RNase-free water and RNA purity and concentration was measured on an Agilent Bioanalyzer (Agilent Technologies, Palo Alto, CA). A total of 200–500 ng of total RNA was used to prepare the RNAseq libraries according to manufacturer’s instructions for the Illumina TruSeq RNA kit (Illumina Inc., San Diego, CA) and sequenced in a single pass 50bp (1x50bp) run on the Illumina HiSeq2000 platform at the University of Colorado’s Genomics and Sequencing Core Facility. Sequencing was done at a sufficient depth to ensure the detection of abundant to moderately rare transcripts.

A custom computational pipeline consisting of the open-source gSNAP, Cufflinks, and R was used for alignment and discovery of differential genes [24–28]. Each sequence generated for each sample was mapped to the human genome (hg19) by gSNAP (version 2012-07-20), and transcript abundance (FPKM) derived by Cufflinks (version 2.2.0). From this, we determined significant gene expression using ANOVA in R (version 3.0.2) between pairwise comparisons of 4 treatment groups: control (untreated), cytomix treated, HTS, HTS+cytomix. Genes with an adjusted p-value of 0.05 (Benjamini-Hochberg correction within the FDR module) were imported into the Ingenuity Pathway Analysis (Qiagen, Redwood City, CA) to identify pathways, molecules, networks, and upstream elements of interest that may be differentially regulated by HTS treatment either alone or in the presence of cytomix. All raw sequence data and ancillary analyses are deposited in the GEO database under the accession GSE94518.

### RNA and protein validations

Total RNA was extracted and two-step qPCR reactions were performed. mRNA in each sample were normalized to the GAPDH and expressed as fold change over the control sample. Supernatants were harvested and a CCL5 ELISA (R&D Biosystems, Minneapolis, MN) was done using 100uL of cell supernatant per well. All experiments were done in triplicate with data represented as means +/-SEM, and analyzed using ANOVA and Tukey’s post–hoc multiple comparison test in GraphPad Prism software (GraphPad Software, Inc., La Jolla, CA).

### Luciferase assay

Changes in the activity of IRF1 and STAT1 were assayed using the Cignal Finder multi pathway dual luciferase reporter array (Qiagen Inc., Germantown, MD). Small airway epithelial cells (SAECS) were transfected with luciferase reporter constructs according to the manufacturer’s protocol. SAECS were co-transfected with an inducible IRF1 responsive construct and a constitutively expressing Renilla luciferase construct. The inducible IRF1 and STAT1 responsive construct encodes the luciferase reporter gene under the control of a basal promoter element joined to tandem repeats of an inducible IRF1 and STAT1 dependent promoter sequences. The inducible reporter monitors changes in activity of IRF1 by measuring the increase or decrease of IRF1 and STAT1 dependent luciferase expression. The constitutively expressing Renilla luciferase reporter expression was used to normalize differences in transfection efficiencies between different treatment groups. A constitutively expressing GFP construct was used for visual confirmation of transfection. Transfected cells were stimulated with cytomix ± HTS for 4 hrs. Changes in promoter activity of IRF1 and STAT1 were assayed by measuring the increase or decrease of IRF1 and STAT1 dependent luciferase expression using the Dual Glo luciferase assay kit (Promega Corp., Madison, WI).

### Whole cell and nuclear extract preparation

P4 SAECS grown in 12-well dishes were treated in the presence and absence of HTS for 2, 4 and 6hrs. At the end of the experiment cells were washed in ice cold PBS and incubated with ice cold RIPA buffer supplemented with protease and phosphatase inhibitor for 10 minutes and centrifuged at 13000 rpm for 10min at 4°C. Cell supernatant was collected and protein concentration was measured using Bradford protein assay. Nuclear extracts were prepared using Nuclear Extract kit (Active Motif Carlsbad, CA) according to manufacturer’s instruction.

### Western blots

25ug of whole cell extract or 30ug of nuclear extract were run on a 4–15% denaturing gradient gel, transferred to a nitrocellulose membrane and analyzed by western blot for p-STAT1, total-STAT1, and IRF1. β-Tubulin and TATA Binding Protein (TBP) were used as loading controls. Bands were detected with west-dura extended ECL detection kit (Pierce Technologies, Rockford, IL). Densitometry and quantitation was performed on a Bio-Rad MP3 gel–doc system.

### Migration assay

SAECS in P3 were cultured in 12-well dishes and stimulated with cytomix with and without HTS for 24 hrs. Supernatants were harvested and applied to the bottom chamber of the chemotaxis plate. The top chamber of the plate was coated with matrigel and seeded with freshly isolated Neutrophil or Splenocyte derived T cells at 5000 cells per well. Plates were incubated 37°C in an Incucyte live cell imaging chamber (IncuCyte^®^ ZOOM System, Essen BioScience, Ann Arbor, MI). Live cell images of the migration of the cells through the matrigel were taken at 2 hr intervals. The data from 5 replicates were compiled and kinetic curve of the chemotaxis of cells from the top chamber towards the chemoattractant in the bottom chamber were generated using onboard software (Essen BioScience).

## Results

### HTS attenuates immune response

**Fig 1:**
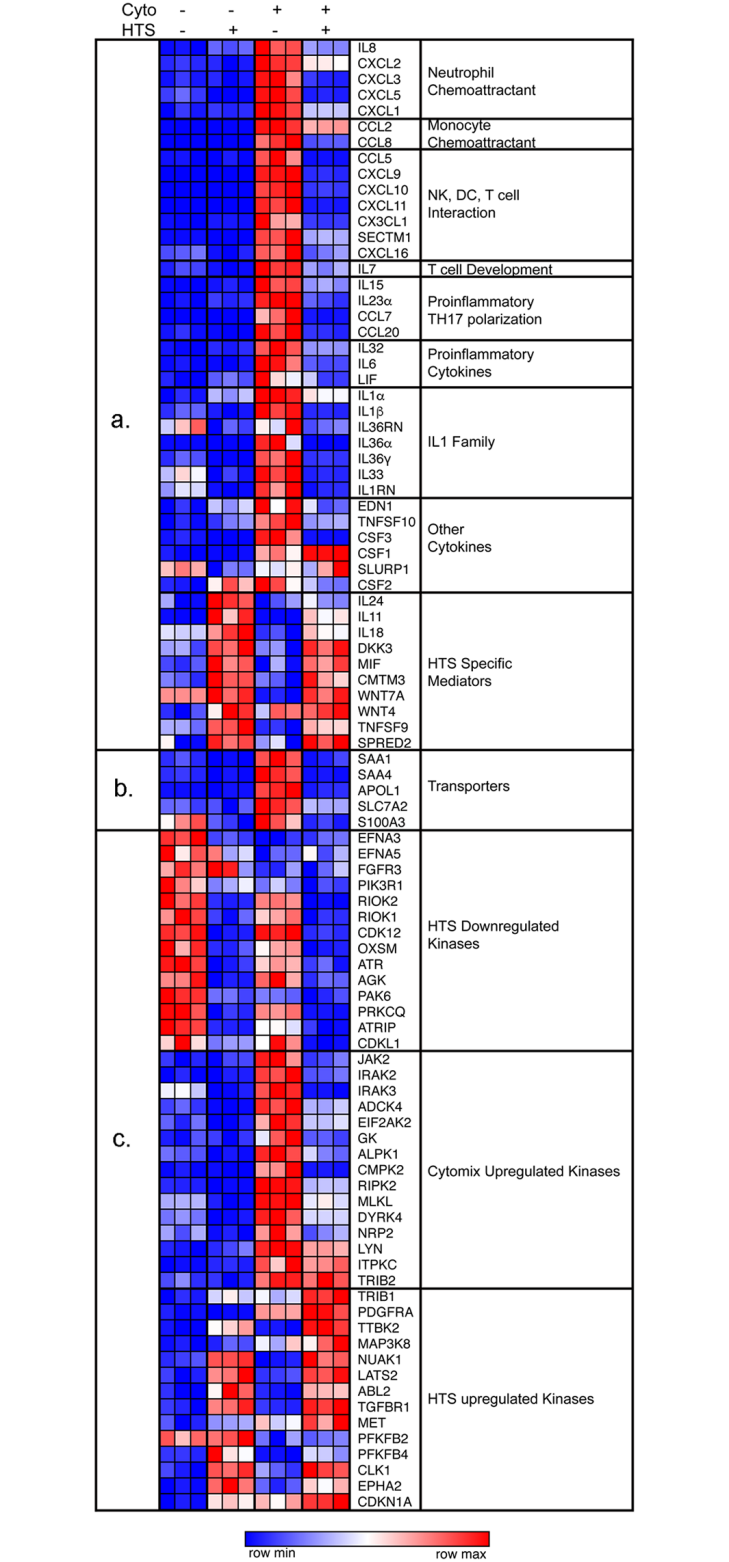
HTS inhibits the expression of several key inflammatory mediators. SAECS in P3 were grown in triplicate to 80% confluence in 12-well dishes, stimulated with TNFα, IL-1β, and IFNγ (cytomix) in the presence and absence of hypertonic saline (HTS) for 4 hrs. RNA was harvested and RNAseq performed. HTS decreases the expression of (a) key chemokines and cytokines, (b) Transporters and (c) Kinases which are important effectors of the inflammatory signaling cascade.

RNA sequencing produced a total of 547.4M reads in total. With a 99% yield after QC and a 82–84% mapping rate, there were an average of 37.7M reads per sample mapped to the genome (S1 Table). Our results show many inflammatory genes significantly upregulated (adjusted P < 0.05) when cytomix was applied without HTS; however, a reduction in these same inflammatory responses was seen when HTS was applied prior to treatment (Fig 1, S2 Table). The transcriptional profile of these cells show that HTS attenuates the expression of many cytokines and chemokines that are induced by cytomix stimulation (Fig 1a). These inflammatory mediators shape both innate and adaptive immune responses via recruitment of effector cells such as neutrophils, monocytes, APC, NK cells, and T and B lymphocytes. The data also illustrate that in addition to diminishing the expression of several key chemokines and cytokines HTS also attenuates the expression of several transporters such as SAA1, APOL1 and SLC7A2 (Fig 1b) and kinases that are induced by cytokine stimulation (Fig 1c).

### Functional validation of immune response

#### HTS significantly downregulates the expression of key chemokines

**Fig 2:**
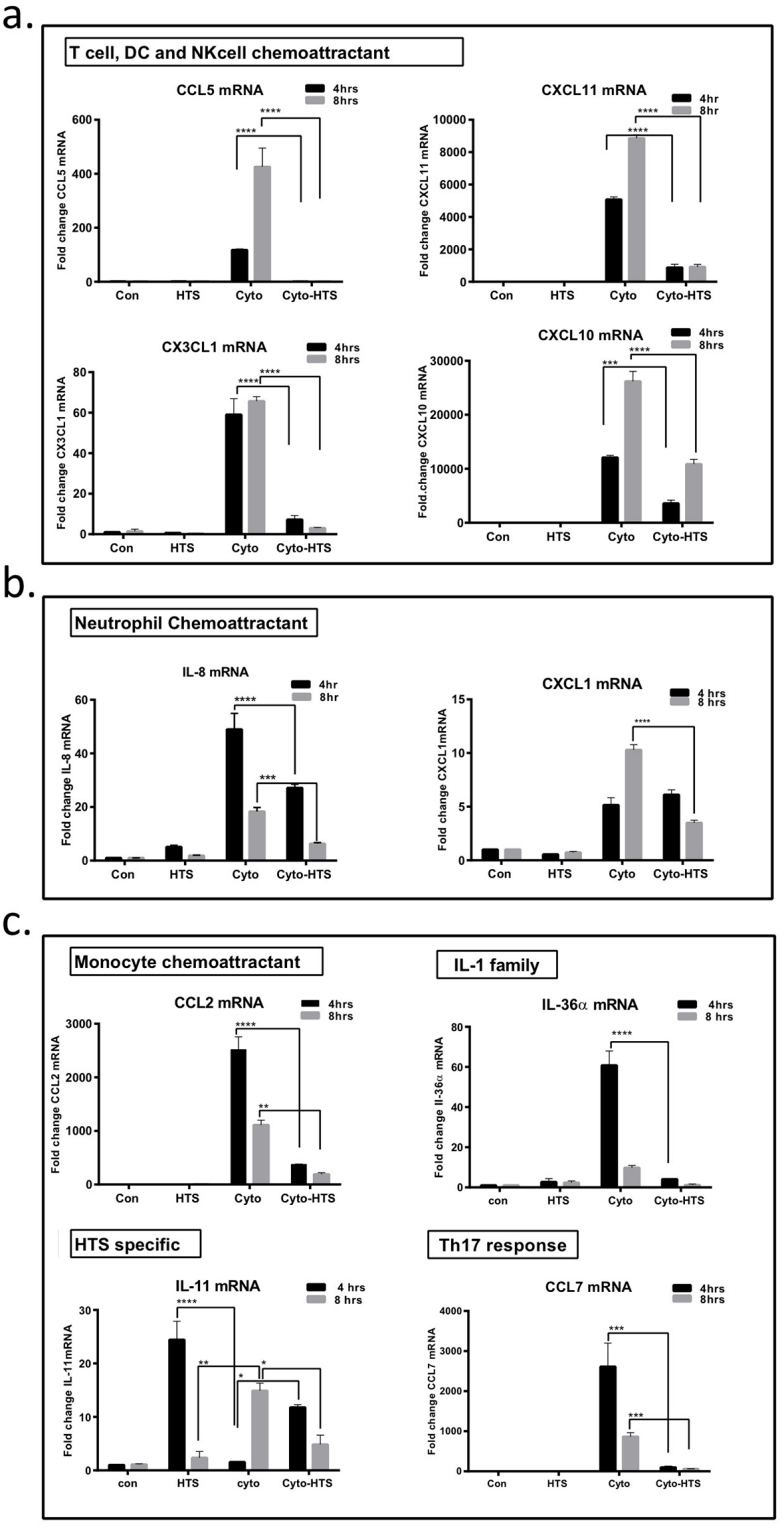
HTS inhibits the expression of chemokines and cytokines that regulate immune cell trafficking and polarization. SAECS in P4 were treated with cytomix in the presence and absence of HTS for 4 and 8hrs, and RNA isolated for qPCR analysis. Validation of the RNAseq shows that (a) chemokines that affect T-cell, Dendritic cell, and NK cell interaction/migration, (b) monocyte trafficking, (c) IL-1 family cytokines, and Th17 response are all increased due to cytokine treatment, and attenuated by HTS; whereas HTS-specific genes such as IL-11 are upregulated in response to HTS. (*p< 0.05; **p< 0.01; ***p< 0.001; ****p< 0.0001).

To validate the RNAseq data, SAECS in P3 were grown to 80% confluence on 12-well tissue culture dishes and treated with cytomix ± HTS for 4 and 8 hrs, respectively. Changes in the expression of some representative chemokines from each subgroup of inflammatory mediators (Fig 1a) were monitored by qPCR. These results also show that the cytokines continue to increase the expression of these released mediators both at 4 and 8hr, whereas the addition of HTS attenuates this response by decreasing the expression of these genes (Fig 2a, 2b and 2c).

#### HTS mediated inhibition of CCL5 correlates to decreased transcriptional activity of IRF1 and STAT1

**Fig 3:**
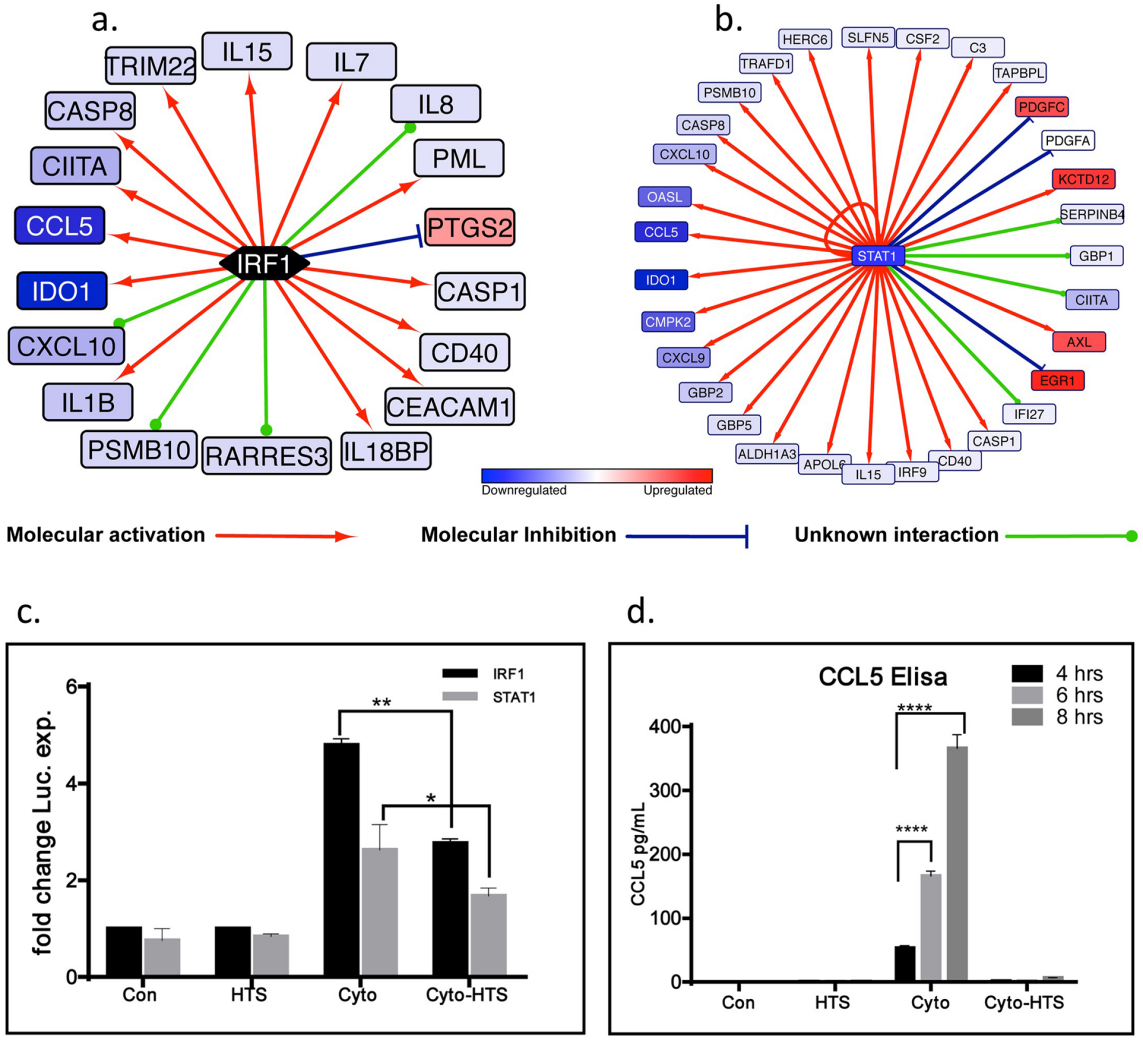
HTS inhibits the transcriptional activation of IRF1 and STAT1 dependent genes. In the Ingenuity Pathway Analysis of cytomix vs cytomix+HTS, IRF1 and STAT1 were inferred to regulate the expression of several downstream molecules in this study. (a) 12 of 18 genes have expression consistent with upstream inhibition of IRF1 by HTS (*Z-score*: *-2*.*564; p = 1*.*05E-10*). (b) 26 of 32 genes have expression consistent with STAT1 inhibition when cytomix is added to cells treated with HTS (*Z-score*: *-4*.*638; p = 3*.*85E-16*). (c) HTS attenuates cytomix-induced luciferase expression in SAECS transiently transfected with IRF1 and STAT1 reporter expression plasmids treated with cytomix for 4hrs in the presence and absence of HTS. (d) The expression of CCL5, a downstream endogenous target of IRF1 and STAT1, is also attenuated by HTS at 4, 6 and 8hrs as determined by ELISA. (*p<0.05; **p<0.01; ***p<0.001; ****p<0.0001).

An upstream transcription factor analysis using the IPA software enumerated IRF1 and STAT1 to be among the top 10 transcription factors (*STAT1 Z-score*: *-4*.*638; p = 3*.*85E-16*, *IRF1 Z-score*: *-2*.*564; p = 1*.*05E-10*) that could differentially regulate the expression of more than 15 different genes whose expressions were significantly altered by HTS treatment in the presence and absence of cytomix (Fig 3a and 3b). Since the CCL5 promoter is under transcriptional control by IRF1 and STAT1, and its expression was significantly decreased (-23.1 fold, p_adj_<1.91E-05) by cytomix+HTS relative to cytomix alone, we assessed the direct effects of HTS on the functional activation of these two transcription factors. A dual luciferase reporter assay was used to interrogate the effects of HTS on the transcriptional activity of IRF1 and STAT1 in SAECS treated with cytomix in the presence and absence of HTS. SAECS transfected with inducible IRF1 and STAT1 reporter constructs show an increase in luciferase expression following cytokine stimulation for 4hrs (Fig 3c). HTS attenuates this response and reduced the expression of the luciferase reporter in both IRF1 and STAT1 reporter constructs. These data were also confirmed at the protein-level by ELISA (Fig 3d).

#### HTS inhibits the chemotaxis of leucocytes

**Fig 4:**
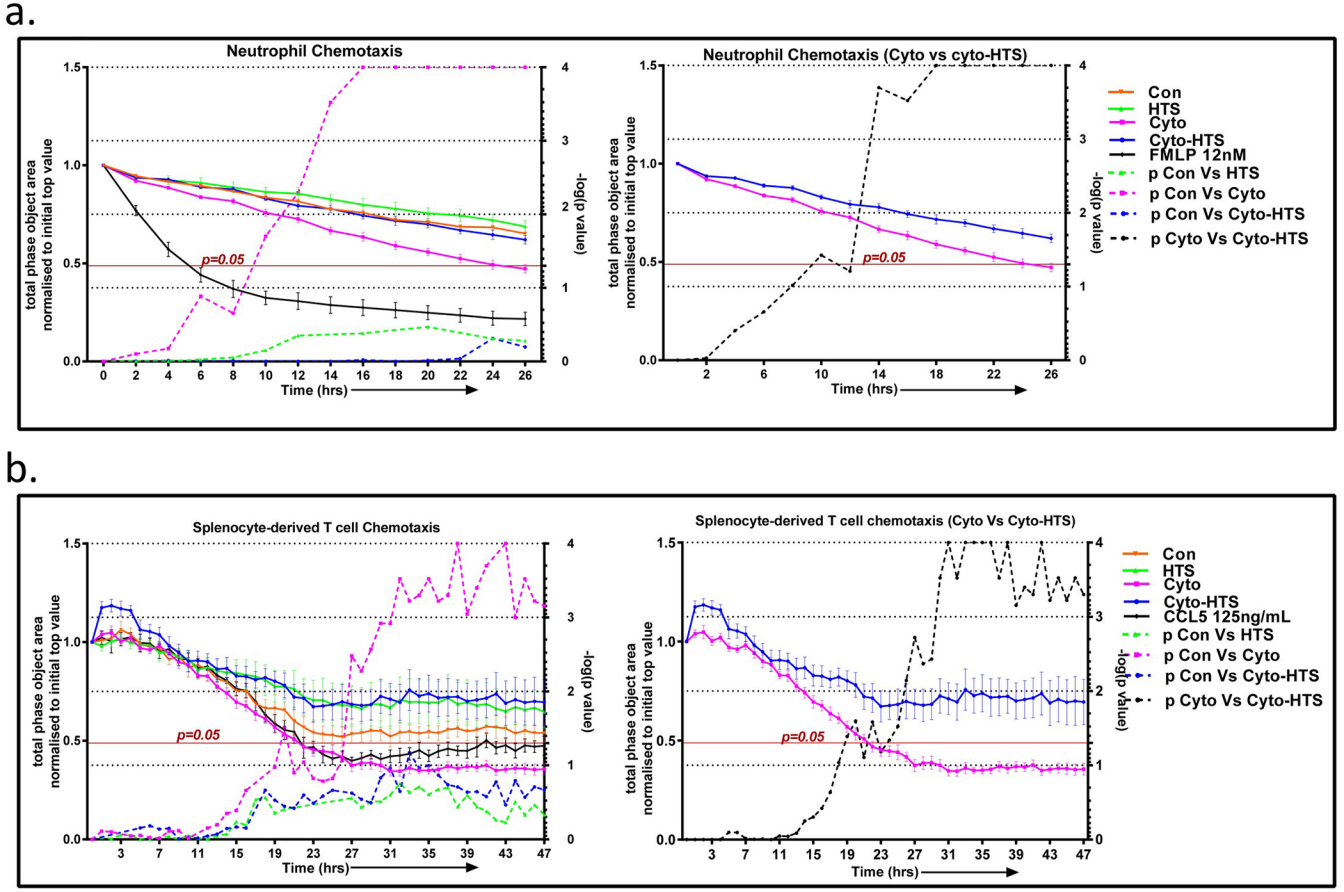
HTS inhibits chemotaxis of neutrophils and splenocyte-derived T-cells. SAECS in P3 were cultured for 24 hrs with cytomix in the presence and absence of HTS and the supernatants were harvested and applied to the bottom chamber of the chemotaxis plate. Chemotaxis of neutrophils and splenocyte derived T cells towards the chemotactic gradient was assayed using Incucyte Zoom imaging system. The results show (a) supernatant from cytomix treated cells elicit maximal migration of neutrophils, and this migration was attenuated in supernatants from cytomix + HTS treated cells. Similarly, in a separate experiment (b), HTS inhibited the migration of splenocyte derived T-cells through the matrigel towards the chemotactic gradient. [solid lines indicate the loss of surface area previously occupied by the neutrophils or splenocytes derived T-cells on the top of membrane as they migrate to the reservoir containing the chemoattractant at the bottom of the membrane; dashed lines indicate p values for pairwise comparison of migration of neutrophils or splenocyte at each time point (control vs HTS, control vs cytomix, control vs cyto-HTS: Fig 4a and Fig 4b main panel; cytomix vs cyto–HTS: Fig 4a and Fig 4b inset). FMLP and CCL5 were used as positive control for the migration assays for neutrophil and splenocyte derived T cells respectively.

A chemotaxis assay was performed to determine if cell supernatants from the SAECS treated with cytomix in the presence and absence of HTS show differential chemoattractant properties for PMNs and splenocyte derived T-cells. SAECS in P3 were treated with cytomix ± HTS and supernatants were collected at 24 hrs. The supernatants were applied to the bottom of a chemotactic chamber. The migration of PMNs and splenocyte derived T-cells towards the supernatant was assayed in real time using the Incucyte Zoom imaging system. Supernatants from cytokine-treated SAECs showed increased migration of both PMNs and splenocyte derived T-cells, and this chemotactic response was inhibited by HTS (Fig 4a and 4b).

### Anti-inflammatory effects of HTS are seen several hours post cytomix stimulation

**Fig 5:**
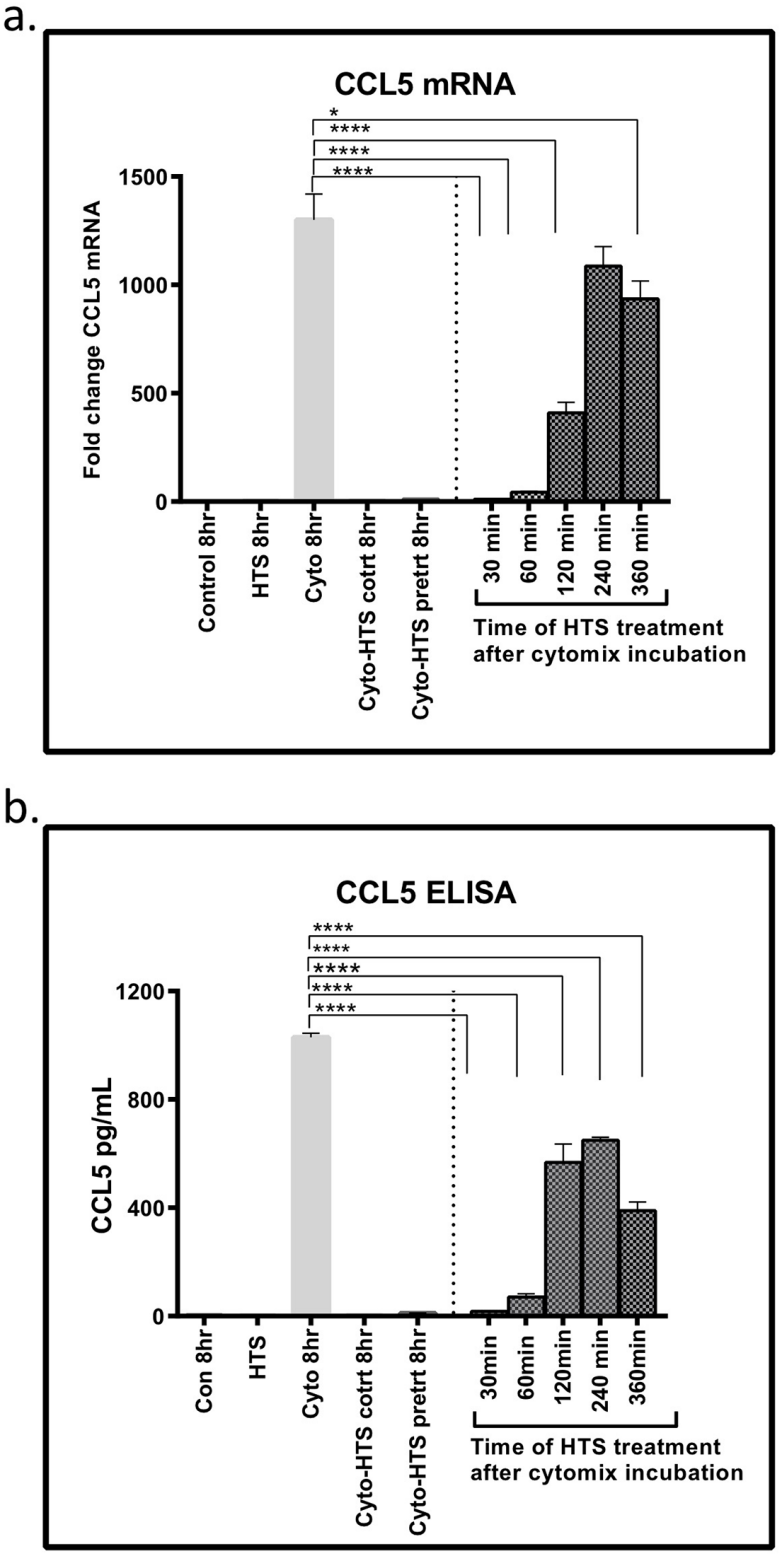
HTS inhibits CCL5 expression in cytokine-pretreated cells. SAECS in P3 were co-treated or pretreated with cytomix ± HTS for 8hrs and HTS was added at 30, 60, 120, 240, and 360 minutes after cytokine addition. The data shows (a) HTS inhibits the expression of CCL5 mRNA and (b) protein in both pre-treatment and co-treatment, as well as when HTS is applied in incremental time points after cytomix pretreatment. Although maximal inhibition was seen when HTS was applied 30mins or earlier after cytokine pre-incubation, the inhibitory effect remains significant when applied even 6 hrs after initial cytokine challenge. (*p<0.05; **p<0.01; ***p<0.001; ****p<0.0001).

Our data show that HTS can prevent inflammatory response when given as a pre-treatment, however, it is not known if HTS can attenuate a preexisting inflammatory response. To determine the effect of timing on dosage, SAECS were treated with HTS at 30mins, 2hrs, 4hrs, and 6hrs post cytokine activation. HTS reduced the expression of CCL5 mRNA and protein at all time points tested (Fig 5a and 5b). However, the anti-inflammatory effects of HTS progressively decreased over the period of inflammatory challenge with maximal effects observed at 30 mins after cytokine treatment indicating that the anti-inflammatory effects of HTS are most advantageous before or immediately following a pro-inflammatory stimulus (Fig 5a and 5b). Similar trends were observed at the transcript level for several other cytokines, with maximal inhibition at 30 minutes post HTS administration (S3 Fig).

### Anti-inflammatory effects of HTS are reversible

**Fig 6:**
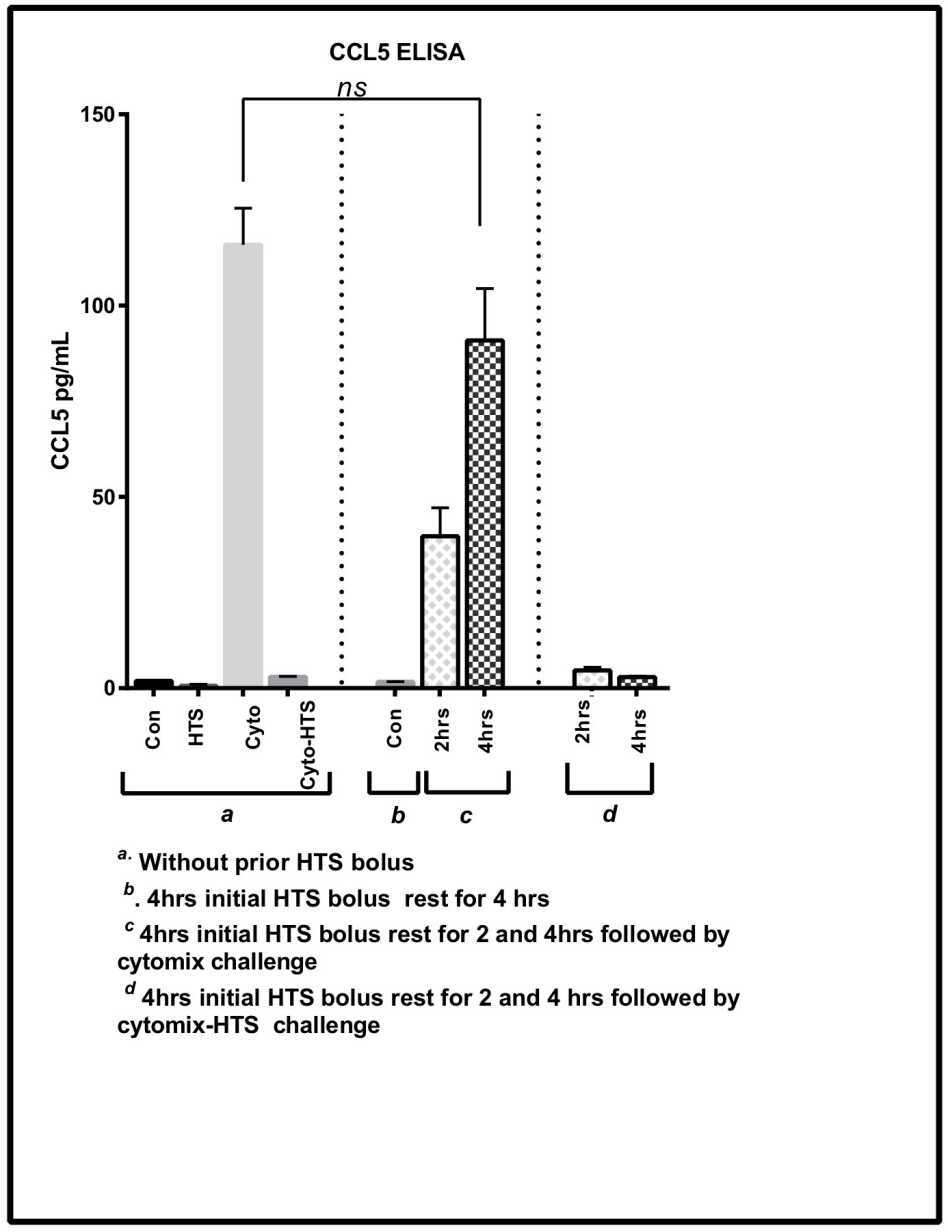
The anti-inflammatory effects of HTS on CCL5 expression are reversible. SAECS in P4 were cultured in the presence of HTS for 4hrs and allowed to rest for 2 and 4hrs in isotonic media before being subjected to a subsequent cytomix challenge. Cells previously treated by HTS can respond to a subsequent cytokine challenge after a minimal recovery period (2-4hrs), with CCL5 levels comparable to initial controls treated with cytomix.

To test if the effects of HTS are reversible, we took previously HTS treated cells and allowed them to rest in isotonic media followed by a cytomix and cytomix-HTS challenge. Fig 6 shows that cells previously treated with HTS can recover and respond to a second inflammatory challenge after 4hrs of rest in isotonic media, indicating that the HTS response is not permanent, and inflammatory responses are subsequently inducible as necessary with the reversibility effect seen after only 2-4hrs of rest in isotonic media.

## Discussion

### HTS can exert potent anti-inflammatory effects

Acute lung injury (ALI) can be the result of major trauma and may proceed to multi-organ failure (MOF) [2, 3, 29]. ALI remains a significant cause of morbidity and mortality in the ICU and may be caused by sepsis, shock, severe extrathoracic trauma, and multiple transfusions following a traumatic event. ALI may progress to acute respiratory distress syndrome (ARDS) and then to respiratory failure with severe pathophysiological consequences leading to fibrin deposition and thickening of the lung interstitial tissue, decreased lung compliance, and recruitment of leucocytes, primarily neutrophils, to the alveolar spaces [6, 7].

A maladaptive immune response is central to the pathogenesis of ALI, which often starts with the activation of the innate immune response via ligation of microbial products or cell injury-associated endogenous products to pattern recognition receptors on the surface of alveolar epithelium [6, 29]. The function of lung epithelium as part of innate immunity is well recognized. The pulmonary epithelium expresses a wide variety of immune response genes such as class I and class II MHC molecules, costimulatory molecules, chemokines, cytokines and prostaglandins following a pro-inflammatory response [7, 30, 31]. HTS may serve as a resuscitative fluid and an immunomodulator as it reduces the synthesis of pro-inflammatory mediators, decreases alveolar macrophage activation, PMN priming and migration to lung tissue both in-vitro and in animal models [8, 9, 15, 16, 18, 21, 32, 33].

#### HTS attenuates trafficking of innate immune cells and can shape the polarization of T-cells

Cytokine stimulation of SAECS activated innate immunity by increasing the expression of several chemokines, including: CXCL1 (Gro-α), CXCL5 (ENA-78), CXCL2 (Gro-β), CXCL3 (Gro-γ) and IL-8 (S2 Table, Fig 1a). These chemokines induce PMN migration and trafficking to site of injury/inflammation [34]. Once they arrive at a site of local inflammation, primed PMNs can do several things: 1) they can phagocytose and destroy infective particles or necrotic cells through engagement of the pathogen recognition receptors such as RAGE and TLRs; 2) PMNs can themselves synthesize several pro-inflammatory mediators and initiate a feed-forward loop that will recruit more neutrophils and other innate effectors such as monocytes, resident tissue macrophages, and NK cells to the inflammatory foci; and 3) PMNs can release alarmins such as HMGB1, defensins and S100 proteins, which link the innate and adaptive responses [4, 35].

HTS also attenuates the cytokine-induced overexpression of chemokines such as CCL2 (MCP1), CCL7 (MCP3) and CCL8 (MCP2) that are effective monocyte chemoattractants (S2 Table, Fig 1a) which are released by airway epithelium and activate monocytes to produce pro-inflammatory cytokines, such as IL-6, and IL-8 [36]. Similarly, chemokines CXCL11 (I-TAC), CXCL9 (MIG), CX3CL1 (FRACTALKINE), CCL5 (RANTES), CXCL10 (IP10) SECTM1, CCL20 (MIP3α), CXCL16 and IL-7 induced by cytomix were attenuated by HTS (S2 Table, Fig 1a). These chemokines can influence adaptive immune responses which promote maturation and survival of T-cells, recruitment of T cells to sites of inflammation [37, 38], as well as promote the polarization of Th1 phenotype [34, 39] promoting T-cell/DC interactions [40] (S2 Table, Fig 1a).

#### HTS attenuates expression of IL-1 family members

HTS also attenuates cytokine-stimulated release of several members of the IL-1 superfamily (S2 Table, Fig 1a). This family of cytokines are major mediators of the innate immune response [41] and propagate the inflammatory response by binding to specific cell surface receptors [41]. IL-1α is a classic endogenous pyrogen that causes increased leucocyte migration to sites of inflammation and can prolong the life span and stimulate the effector function of PMN [41]. IL-1αand IL-33 can also act as “alarmins” in response to tissue damage and serve as a critical link between the innate and adaptive immunity by promoting uptake of antigens by immature DC and their subsequent presentation to naïve T cells and increasing expression of Th2 cytokines IL-5 and IL-13 [42–44]. Thus, these alarmins are likely a possible therapeutic target to both dampen inflammation and to uncouple the innate and adaptive responses [42, 45]. The IL-36 response mirrors the effects of IL-1 and may serve as an amplifier of the innate immune response and a mediator of pulmonary inflammation and fibrosis [46].

#### HTS down regulates expression of chemokines that promote Th17 phenotype

Cytokine stimulation also increased the expression of IL-23, CCL7, and IL-15 (S2 Table, Fig 1a), which are known to induce the differentiation of naïve CD4+ T-cells to a highly pro-inflammatory IL-17-producing Th17 phenotype [34, 47–49]. IL-17 in turn induces T-cell priming, activation of lung fibroblasts, activation of endothelial and epithelial cells to produce multiple pro-inflammatory mediators, including IL-6, TNF-α, NOS-2, MMPs and chemokines (IL-8, CXCL1), growth factors (CSF3, CSF2) and adhesion molecules (ICAM1), which further exacerbate the airway inflammatory cascade via PMN recruitment to the lung [47, 48, 50].

Additionally, HTS attenuated the cytokine-induced increases in acute phase mediators IL-6 and LIF (S2 Table, Fig 1a and 1b). Prolonged activation of these molecules correlates to increased PMN infiltration into the lung [51], which is deleterious in the setting of chronic inflammation and the systemic inflammatory response. HTS also inhibits the expression of transporters such as serum amyloids SAA1 and SAA4 (S2 Table, Fig 1a and 1b), now recognized as major players in the acute phase reaction. SAAs have strong immunological properties where they can activate the inflammosome cascade and induce the synthesis of several cytokines. They are also chemotactic for PMNs and T-cells, which is a key step in ALI and represents a possible therapeutic target to inhibit leucocyte migration to the lung [52].

#### HTS affects the expression of MAP kinases

HTS attenuates the expression of several non-receptor serine, threonine, and tyrosine kinases (e.g., JAK2, LYN,and IRAKs) whose expression is increased by cytomix treatment (S2 Table, Fig 1c). Their role in adaptive and immune response is well documented. LYN is a tyrosine kinase involved in cellular responses to cytokines, regulation of MAPK signaling, initiation of the B cell response and differentiation and modulation of cell survival and apoptosis [53, 54]. IRAK2 and IRAK3 are kinases of the interleukin receptor signaling pathway and participate in IL-1-induced increases of NF-κB dependent pro-inflammatory cytokines [55]. JAK2 is a member of the Janus kinase family of non-receptor tyrosine kinase and is involved in IL6 family cytokine signaling through gp130/JAK STAT pathway [56]. Downstream targets of the JAK/STAT pathway include transcription factors such as IRF1 and CEBPδ that are known to activate the transcription of pro-inflammatory mediators such as CCL5, a T-cell chemoattractant. HTS attenuates the expression of most of these kinases, thereby inhibiting the downstream expression of several molecules that propagate the inflammatory response [52].

There are also HTS specific effects which are independent of cytomix challenge. Results of the RNA seq analysis and validation studies show that IL-11 mRNA expression was increased over 10 fold by HTS alone. Additionally, we observe cytokine independent effects of HTS. For example, HTS independently increases the basal expression of proteins such as DKK3 [57], CMTM3 [58], MIF [59] and CDKN1A [60] that regulate cell cycle progression and apoptosis, and mediates inhibition of cell proliferation (Fig 1c). This proliferative inhibition is likely a manifestation of the initial cellular stress response to a hyperosmolar environment; this and the effects of HTS on cell cycle regulation have been detailed separately [61]. Ultimately, our data elucidates that HTS can exert potent anti-inflammatory effects by inhibiting the expression of several key inflammatory mediators with less than 10% apoptotic induction between the different treatment conditions.

### Anti-inflammatory effects of HTS are cell type and stimulus specific

HTS-mediated inhibition of pro-inflammatory mediators is highly stimulus and cell type specific. The specificity of stimulus and cell type also determines the choice of transcription factors regulating the expression of these pro-inflammatory mediators. For example, in A549 pulmonary type II alveolar lung carcinoma cells, HTS was able to inhibit the synthesis and release of NF-κB dependent chemokines CCL5 and CXCL10, in response to both TNF-α and IL-1β, but it selectively inhibited the synthesis of the NF-κB dependent gene CCL2 in response to TNF-α, but not to IL-1β [9]. In contrast, in the present study NF-κB was not identified among the top 10 transcription factors affected by HTS. Rather, HTS inhibited the activation of two key transcription factors (STAT1, Z-score: -4.638; p = 3.85E-16 and IRF1 Z-score: -2.564; p = 1.05E-10) that further regulate other groups of genes including the chemokine CCL5 (Fig 3a and 3b). Thus, we further examined the possible mechanisms by which HTS mediates STAT1 and IRF1 activity. Although reduced IRF1 nuclear localization can inhibit expression of IRF1 dependent downstream targets, we did not observe any significant difference in levels of nuclear IRF1 localization due to HTS (S3 Fig). Additionally, as the IRF1 promotor is under the transcriptional control of STAT1, and phosphorylation of STAT1 at Ser-727 is necessary for its optimal transcriptional activity, it is possible that the inhibition of IRF1 transcriptional activation is due to differential STAT1 phosphorylation; however, we do not see a significant difference in STAT1 phosphorylation at 2, 4 and 6 hrs (S4 Fig). Conversely, RNA extracted from these same cells show a significant difference in STAT1 mRNA levels at 2 and 4 hrs (S4 Fig). This suggests that even though HTS did not alter STAT1 activation status, it inhibited STAT1 and IRF1 downstream responses by inhibiting STAT1 mRNA synthesis. Both these data suggest that HTS mediated inhibition of IRF1 and STAT1 is a complex process and a comprehensive analysis of upstream regulation of the transcription modules are required to fully dissect the mechanism of HTS mediated inhibition of inflammation. Based on pathway analysis, we have identified two candidate molecules that may contribute to HTS mediated inhibition of STAT1 transcription. TRIM24 is a pleiotropic protein that has been identified as a negative regulator of STAT1 [62]. In the current study, the predicted activation status of TRIM24 (Z-score: - 4.577; p = 4.03E-17) suggests that it is highly activated in cells stimulated with cytomix-HTS, and thus could be a potential upstream regulator of HTS-mediated STAT1 transcriptional regulation. Another potential candidate that could regulate STAT1 expression is the epigenetic protein CREBBP (CREB Binding Protein). This nuclear protein is a transcriptional coactivator of the cAMP response element binding protein (CREB) which was also identified as one of the upstream effector molecules (Z-score: - 3.037; p = 2.89E-13) strongly activated in cytomix/HTS treated cells. Kramer et al. have shown that CREBBP can regulate STAT1 signaling through CREBBP -mediated STAT1 acetylation [63]. Future studies will involve experiments that will explore mechanisms of HTS-induced inhibition of the inflammation and the role of upstream transcriptional regulators such as TRIM24, CREBBP and CREB in this process.

### Clinical relevance of HTS as a therapeutic

The therapeutic benefits of HTS in CF are well documented. Aerosolised 3% HTS or higher concentrations have been used improve mucociliary clearance in CF patients by increasing the tonicity of Airway Surface Liquid and creating an osmotic gradient that would draw water into the airway and facilitate the removal of mucous [64]. Recent studies in CF patients have extended some of the beneficial effects of HTS to its anti-inflammatory properties and its ability to prevent harmful effects caused by increased neutrophil infiltration into the lungs of CF patients [65]. Our in-vitro data is complementary as we show that HTS exerts strong anti-inflammatory effects in cytomix challenged airway epithelial cells and prevents neutrophil chemotaxis. The anti-inflammatory effects of HTS and the fact that it has been well tolerated in CF patients makes it a potentially attractive therapeutic candidate in the treatment of ARDS/ALI in which several pro-inflammatory mediators such as IL-6 and IL-8 are elevated.

Our data suggests that HTS has strong anti-inflammatory effects which is a beneficial effect in the initial proinflammatory phase following injury, which produces collateral damage such as neutrophil infiltration and subsequent lung fibrosis and dysfunction. However, these anti-inflammatory effects are also accompanied by immunosuppression with downregulation of several inflammatory and immune mediators. While the initial proinflammatory phase and its adverse outcomes are prevented, the immunosuppression may leave the host susceptible to subsequent infectious complications and delayed morbidity and mortality. Further studies will be needed to delineate the timing of these anti-inflammatory effects and whether they produce delayed complications using in-vivo animal models. Our initial in-vitro studies in this regard are very encouraging as we show that anti-inflammatory effects of HTS are reversible 24 hrs after an initial HTS exposure (Fig 6). Finally, nebulized hypertonic saline may prove the ideal delivery mechanism, as it should provide local anti-inflammatory effects with avoidance of a systemic immunosuppresion.

## Conclusion

The overarching effect of HTS is to decrease the pro-inflammatory state of the lung and extends to both innate and adaptive immunity. Pro-inflammatory cytokines cause activation of innate immune responses through PMNs and other leukocytes that migrate to the site of injury. These are central components in the initiation of this process and can further amplify this by secreting inflammatory mediators that act as a chemotactic gradient to recruit more leucocytes to the affected sites. Our data show that HTS attenuates this response by functioning as a ‘brake’ and decreasing the expression of several chemokines involved in the progression of innate immune response and ultimately reducing the migration of immune cells, even hours after an inflammatory challenge. Future experiments will extend our present findings and use the physiologically more relevant Air Liquid Interface culture for an in depth analysis of cellular and metabolic effects of HTS in airway epithelial cells and the role of upstream transcriptional modules that may affect downstream anti-inflammatory responses induced by HTS. Subsequent experiments in human and animal models will further interrogate the anti-inflammatory mechanism of inhaled HTS *in vivo* and the determination of the optimal time and dose of HTS administration as an anti-inflammatory therapeutic agent.

## Supporting information

S1 Table RNAseq quality control table. Read counts show a 99% retention after base quality quality control, and an 82–84% mapping rate.
(TIF)

S2 Table Gene list and RNAseq expression data. SAECS stimulated with HTS ± cytomix show differential expression of inflammatory mediators, transporters and kinases. Expression for control (N1, N2, N3); cytomix added (N4, N5, N6); HTS added (N7, N8, N9); and cytomix + HTS samples (N10, N11, N12) are shown.
(TIF)

S1 Fig Cytomix challenged SAECS show a dose response effect to HTS. SAECS pretreated for 4hrs with HTS ± cytomix at 300 mOsm, 400 mOsm and 500 mOsm HTS show significant downregulation of all the inflammatory mediators at 400mOsm HTS.
(TIF)

S2 Fig Anti-inflammatory effects of HTS are observed post cytomix challenge for several other inflammatory mediators. SAECS post treated or cotreated with HTS at incremental time points after cytomix stimulation show significant downregulation of CXCL1, CXCL11, CX3CL1 and CCL2 mRNA.
(TIF)

S3 Fig HTS does not inhibit nuclear translocation of IRF1. Western blot of nuclear extracts from SAECS treated with HTS ± cytomix at 2 and 4 hrs demonstrate that HTS cannot significantly inhibit cytomix mediated nuclear translocation of IRF1.
(TIF)

S4 Fig Effect of HTS on STAT1 protein and mRNA. Western blot and qPCR analysis of STAT1 protein and mRNA show that HTS cannot inhibit STAT1 phosphorylation but can downregulate STAT1 mRNA at 2 and 4hrs.
(TIF)
