# Supplementary figures and images for: Hypertonic saline attenuates the cytokine-induced pro-inflammatory signature in primary human lung epithelia
Source: PLoS One. 2017 Dec 18;12(12):e0189536. doi: 10.1371/journal.pone.0189536 (PMC5734749; doi:10.1371/journal.pone.0189536)

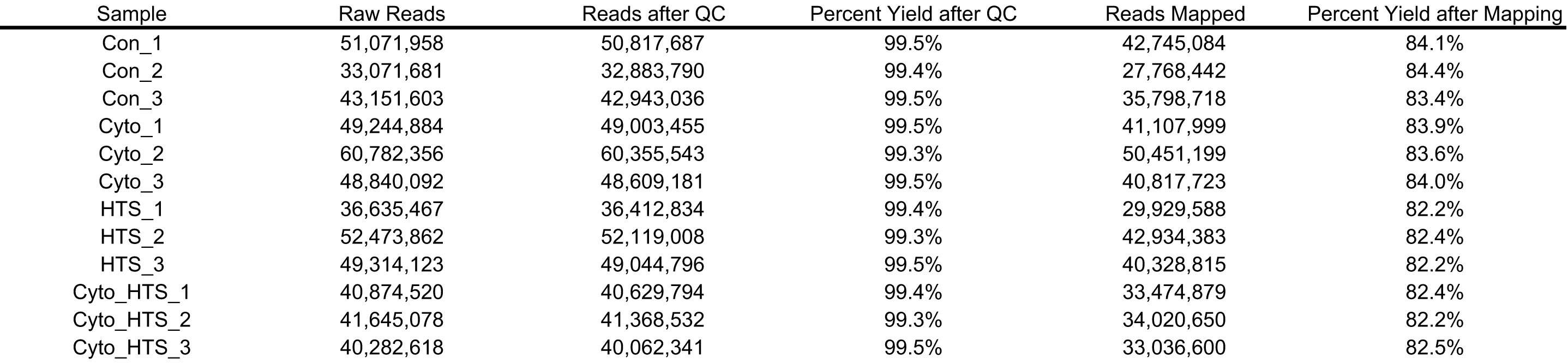

Supplement: S1 Table — Read counts show a 99% retention after base quality quality control, and an 82–84% mapping rate. (TIF) [file pone.0189536.s001.tif]

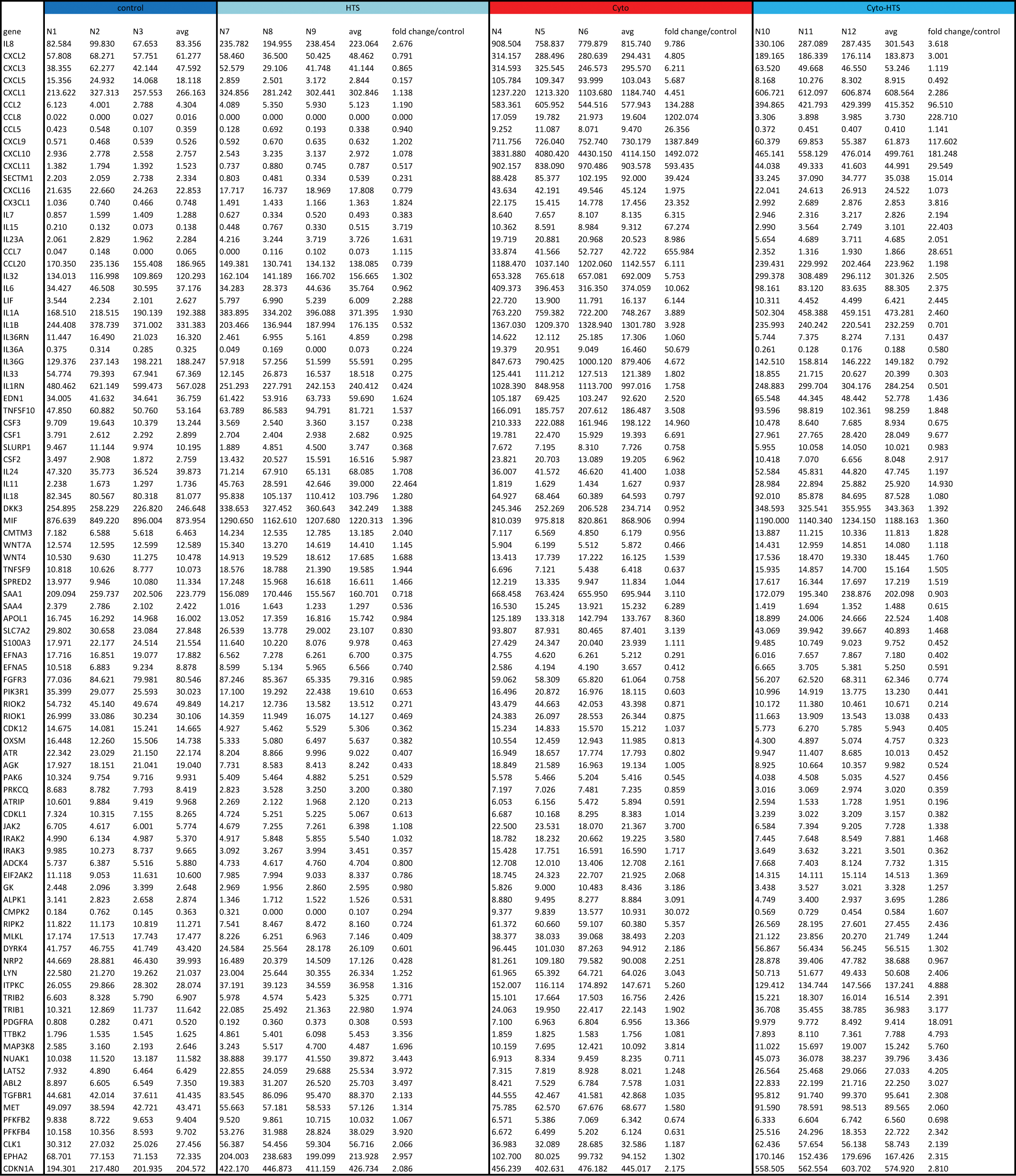

Supplement: S2 Table — SAECS stimulated with HTS ± cytomix show differential expression of inflammatory mediators, transporters and kinases. Expression for control (N1, N2, N3); cytomix added (N4, N5, N6); HTS added (N7, N8, N9); and cytomix + HTS samples (N10, N11, N12) are shown. (TIF) [file pone.0189536.s002.tif]

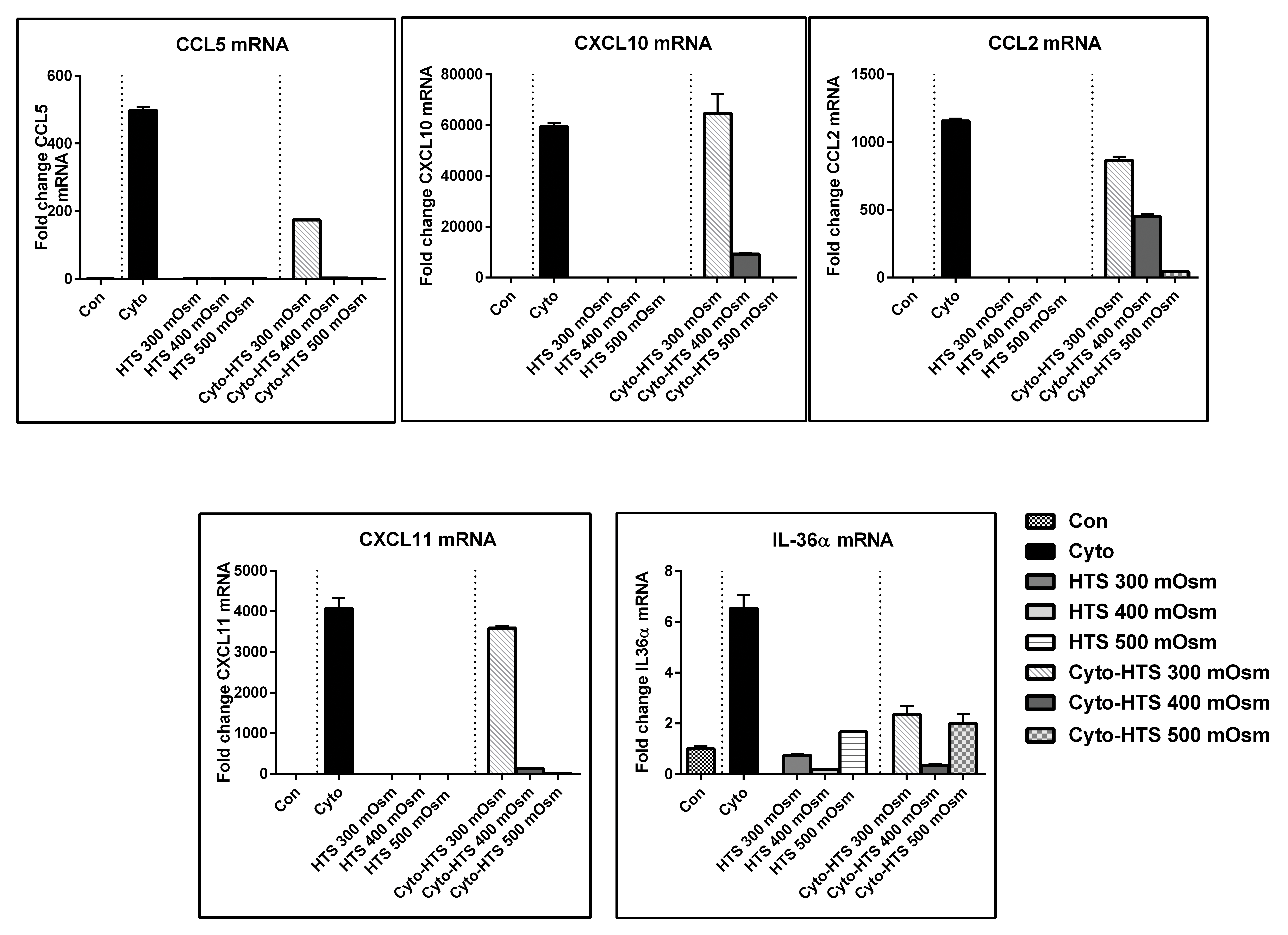

Supplement: S1 Fig — SAECS pretreated for 4hrs with HTS ± cytomix at 300 mOsm, 400 mOsm and 500 mOsm HTS show significant downregulation of all the inflammatory mediators at 400mOsm HTS. (TIF) [file pone.0189536.s003.tif]

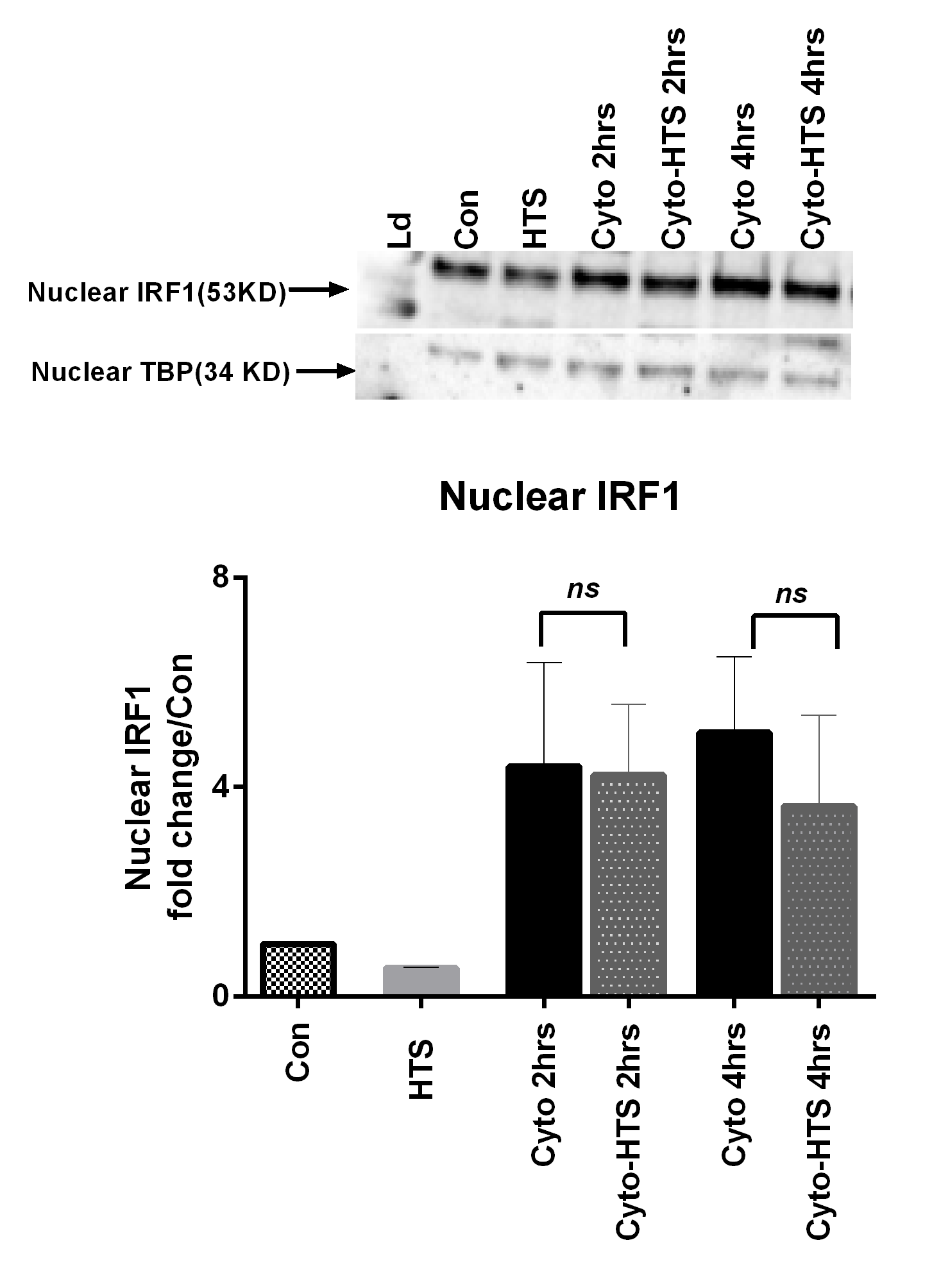

Supplement: S3 Fig — Western blot of nuclear extracts from SAECS treated with HTS ± cytomix at 2 and 4 hrs demonstrate that HTS cannot significantly inhibit cytomix mediated nuclear translocation of IRF1. (TIF) [file pone.0189536.s005.tif]

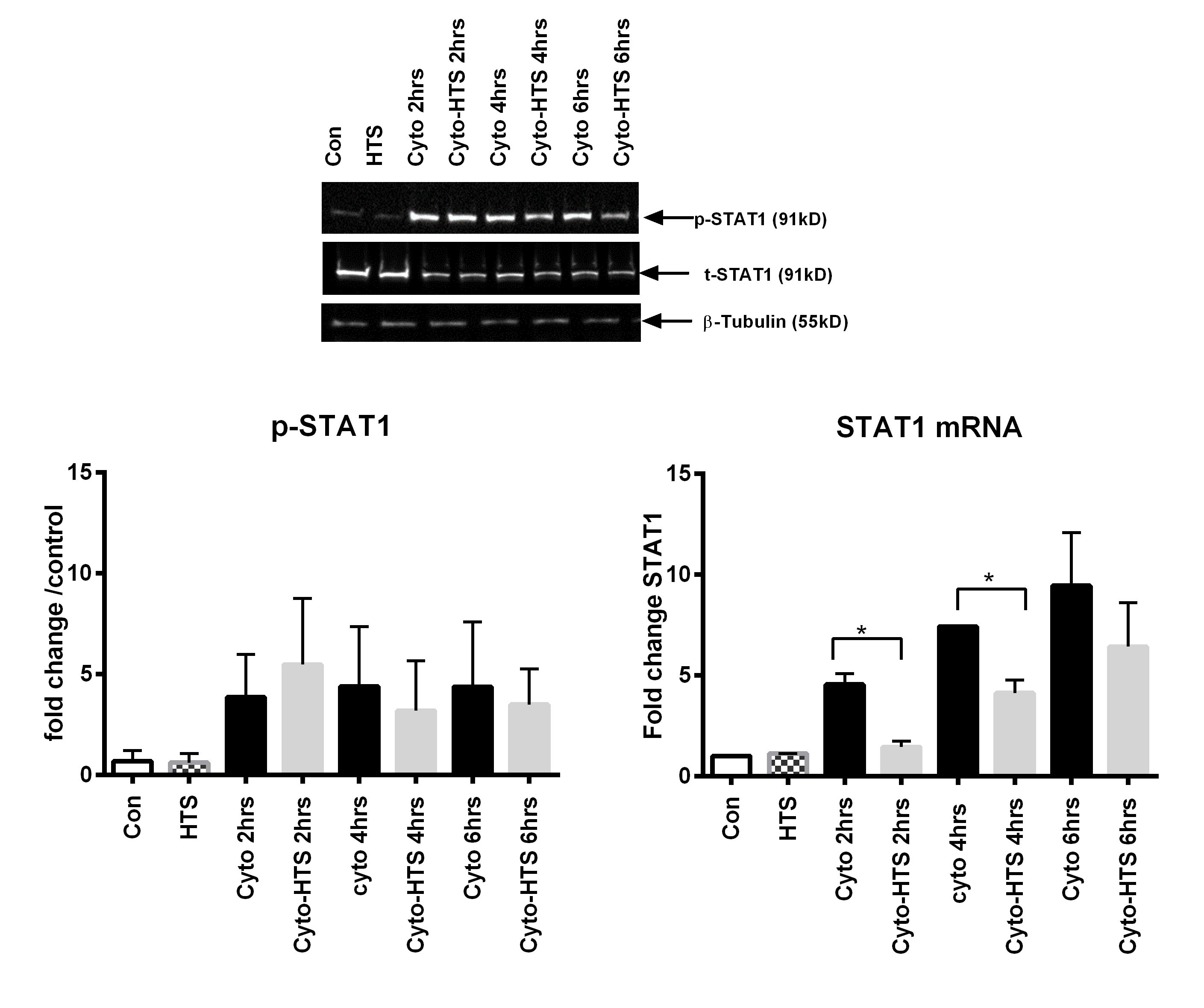

Supplement: S4 Fig — Western blot and qPCR analysis of STAT1 protein and mRNA show that HTS cannot inhibit STAT1 phosphorylation but can downregulate STAT1 mRNA at 2 and 4hrs. (TIF) [file pone.0189536.s006.tif]
